# Transcriptional Regulatory Network of the Embryonic Diapause Termination Process in *Artemia*

**DOI:** 10.3390/genes16020175

**Published:** 2025-02-01

**Authors:** Bin Wang, Zhen He, Mingzhi Zhang, Ruiqi Zhang, Zhentao Song, Anqi Li, Tong Hao

**Affiliations:** 1 Tianjin Key Laboratory of Animal and Plant Resistance, College of Life Sciences, Tianjin Normal University, Tianjin 300387, China; 2 Tianjin Key Lab of Aqua-Ecology and Aquaculture, Fisheries College, Tianjin Agricultural University, Tianjin 300384, China

**Keywords:** *Artemia*, diapause, embryonic pause termination, regulatory network, transcription factor

## Abstract

*Artemia* is a typical animal used for the study of the diapause mechanism. The research on the regulation mechanism of diapause mainly focuses on the occurrence and maintenance of diapause. There are few studies on the mechanism of embryonic pause termination (EDT), especially for its transcriptional regulation mechanism. This study integrated transcriptional regulatory data from ATAC-seq and gene expression data from RNA-seq to explore the transcriptional regulatory mechanisms involved in the EDT process. Through integrated analysis, four important transcription factors (TFs), SVP, MYC, RXR, and SMAD6, were found to play a role in the EDT process, in which SVP, MYC, and RXR were upregulated, while SMAD6 was downregulated in the EDT stage. Through co-expression analysis, a transcription regulatory network for these four TFs was constructed and the functions of the TFs were analyzed. The expression of the TFs was further verified by RT-qPCR. Through functional analysis, SVP was found to be predominantly involved in cell adhesion and signal transduction. MYC probably played a role in protein binding. RXR may function in the process of RNA binding and the transfer of phosphorus-containing groups. Smad6 regulated the signal transduction, cell adhesion, and oxidation–reduction processes. The expression of the key TFs was verified by RT-qPCR. The results of this work provide important clues for the mechanism of transcriptional regulation in the EDT process of *Artemia*.

## 1. Introduction

*Artemia* is a type of crustacean capable of living in high-salinity environments and serves as one of the essential foods for aquatic animals. Under harsh environmental conditions, their developmental process involves a typical dormancy mechanism, which results in the formation of dormant embryos (cysts) [1]. This mechanism allows *Artemia* to survive in extreme environments such as drought, high salinity, and extreme temperatures. When environmental conditions are unfavorable, the embryos of *Artemia* form cysts externally. These cysts exhibit a strong resistance to extreme temperatures, drought, and high salinity. During diapause, embryonic metabolic activities are nearly halted, and this state can persist for several years. Once the environmental conditions improve, the dormant embryos begin to absorb water and restart their metabolic processes, gradually recovering from diapause. The primary trigger for diapause termination is the recognition of improved environmental conditions. Commonly, under the stimulation of −20 °C and dryness, the cyst will break the diapause and enter the post-diapause stage. In the post-diapause stage, once the cysts recognize appropriate environmental signals, such as suitable temperatures, optimal salinity levels, and the absence of stressors or predators, the diapause state is terminated and embryonic development is restarted. Once the diapause termination is started, it cannot return to the diapause state again. The embryos will continue to develop and hatch into larvae within 24 h, initiating a new life cycle [2]. This unique mechanism makes *Artemia* an ideal model for studying embryonic diapause, biological adaptability, and responses to environmental stress [3]. On the other hand, diapause is a phenomenon with a very wide distribution, observed in plants, animals, and even human cancer cells [4,5,6]. This widespread distribution suggests that diapause likely has ancient evolutionary origins and may share a common molecular basis. Therefore, studying the diapause mechanisms of *Artemia* can also provide valuable insights into the diapause processes of other organisms.

The shell of the dormant embryos of *Artemia* is rich in polysaccharides and proteins, which form a vitrified state under dry conditions, protecting the internal cellular structures and molecules from damage [3]. Additionally, the dormant embryos contain a large amount of protective proteins, such as heat shock proteins and small molecular chaperones, which play a protective and reparative role during embryo revival, preventing protein denaturation and cell membrane damage [7,8,9,10,11]. Researchers have identified several genes and proteins related to embryonic diapause termination (EDT) in brine shrimp. These include, for example, the diapause hormone receptor-like gene (*Ar-DHR*) [12], P53 and DNA damage-regulated gene 1 (*pdrg1*) [13], apoptosis inhibitor 5 (API5) [14], nuclear factor protein Ar-DEK [15], and retinoblastoma binding protein 4 (RBBP4) [16]. Moreover, glycerol kinase has also been found to play a significant role in the EDT of *Artemia sinica* and is highly expressed during early embryo development. Its expression is induced by stresses such as low temperature and high salinity [17].

Some signaling pathways related to EDT have also been gradually discovered. For example, the Wnt signaling pathway has been found to be associated with the EDT in *Artemia* [15]. In addition, in other species, some signaling pathways have also been confirmed to play roles in EDT. For instance, the mTOR signaling pathway has been found to be related to cell dormancy in human cancer cells [18] and *Bactrocera minax* [19]. The Notch signaling pathway has been shown to be involved in both diapause and the EDT process in *Caenorhabditis elegans* embryos [20].

In terms of the study of transcriptional regulation, existing research mainly focuses on diapause embryo formation and diapause maintenance. The transcription factor (TF) p8 was found to regulate autophagy during diapause embryo formation in *Artemia parthenogenetica* [21]. The TF FoxO influences diapause maintenance in *Laodelphax striatellus* nymphs by affecting carbohydrate, amino acid, and fatty acid metabolism, as well as the phosphatidylinositol 3-kinase/protein kinase B signaling pathway. Knocking out FoxO significantly shortened the developmental period of *L. striatellus* nymphs [22]. The circadian TF Vrille may be involved in regulating follicle development arrest in female *Culex pipiens* [23]. In addition, the autophagy gene Sequestosome-1/p62 (*SQSTM*) was upregulated in the diapause cyst of *Acartia tonsa*, which is a gene that functions in the stress response [24]. In *Neocalanus flemingeri*, the genes involved in the 20E cascade pathway, the TCA cycle, and RNA metabolism were upregulated and those in chromatin silencing were downregulated after one hour of EDT [25]. Genes related to oogenesis, RNA metabolism, and fatty acid biosynthesis were upregulated in diapause *Calanus finmarchicus* [26], which partly agreed with the results in *L. striatellus* [22] and *N. flemingeri* [25].

Although some progress has been made in the study of EDT, the specific mechanisms of EDT in *Artemia* are still not well understood. In our preliminary research, we performed ATAC-seq and RNA-seq sequencing and analysis on *Artemia* cysts during the diapause period, 30 min after EDT, and 5 h after EDT. We found that significant changes in gene expression could be detected at the transcriptome level as early as 30 min after the breaking of diapause [27], which was consistent with the findings of Wang et al. [28]. Signal transduction plays a crucial role in the EDT process. Furthermore, there were substantial differences in the signal transduction pathways and GPCR proteins that were active within the first 30 min and from 30 min to 5 h after EDT [29].

The purpose of this study was to investigate the transcriptional regulatory network function in the EDT process of *Artemia*, which may supply a systematic view instead of a single gene or TF for the mechanistic analysis of EDT. Based on the ATAC-seq and RNA-seq sequencing results retrieved from our previous research, this study further identified the TFs active in the EDT process through the integration analysis of these two types of high-throughput data. Additionally, by conducting co-expression analysis, a transcriptional regulatory network was constructed to uncover the regulatory functions of these transcription factors in the EDT process. The findings of this study are of great significance for further exploring the transcriptional regulatory mechanisms involved in the EDT processes of *Artemia* and other organisms.

## 2. Materials and Methods

### 2.1. Design of the Experiments

**Figure 1:**
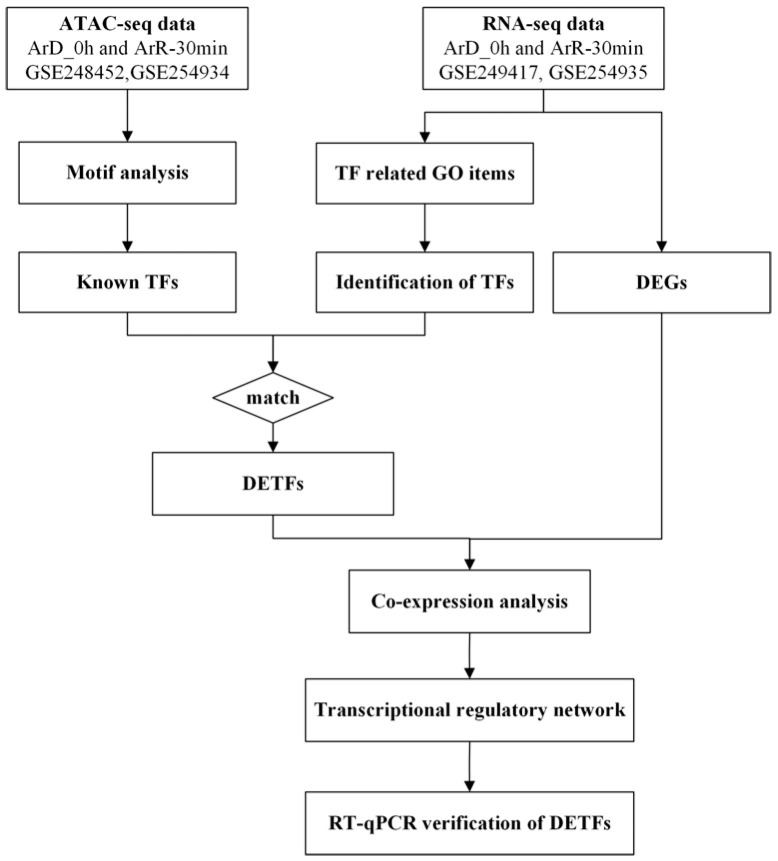
The workflow diagram for this work.

In order to identify the TFs involved in EDT, the ATAC-seq and RNA-seq datasets for 0 h and 30 min after EDT were employed. The potential motifs were predicted based on the ATAC-seq dataset and compared to the motifs of known TFs to identify the possible known TFs existing in *Artemia*. Besides, the differentially expressed genes (DEGs) with TF activity were also identified based on the GO enrichment analysis results from the RNA-seq dataset. The two groups of TFs were compared and the matched genes were selected for mutual verification in order to improve the reliability of the results. The matched genes were defined as differentially expressed transcription factors with significant chromatin binding motifs (DETFs). Subsequently, the co-expression analysis was performed between the DETFs and DEGs to construct the transcriptional regulatory network involved in EDT, and the expression of the DETFs was further validated with RT-qPCR. The workflow diagram is shown in Figure 1.

### 2.2. Motif Analysis Based on ATAC-Seq

A published study investigated the gene expression in *Artemia* after EDT with 0.5 h intervals from 0 to 7 h [28]. They found that the activation of cellular activities started as early as 0.5 h after EDT. In our early study, we also found obvious differences in the gene expression after 30 min of EDT [27], which indicated that the first 30 min recovery period was important for the activation of EDT. Therefore, 30 min was selected as an experimental time point in this study in order to investigate the transcriptional regulation involved in the activation of EDT. The ATAC-seq datasets for the diapause stage and 30 min after EDT in *A. parthenogenetica* were downloaded from GEO database and analyzed. The data numbers for the diapause stage and 30 min after EDT were GSE248452 and GSE254934, respectively, and the samples were designated as ArD_0h and ArR_30min. Three biological replicates were used for each stage in ATAC-seq. Motifs represented the sequence conservation at the location of the peak, which may play a role in gene expression regulation. The sequence information for 250 bp upstream and downstream of the peak summit (a total of 500 bp) was used and Homer software (version v4.11, New Haven, CT, USA, with parameters -gc -len 8, 10, 12, 14) was employed to identify conserved sequence features at peak-enriched locations. The motifs with lengths of 8, 10, 12, and 14 bp were identified for each sample. Motif enrichment analysis was performed against known transcription factor motifs, and those with a *p*-value ≤ 0.01 were considered significant.

### 2.3. Identification of EDT Related TFs

The RNA-seq datasets for the diapause stage and 30 min after EDT in *A. parthenogenetica* were downloaded from GEO database. The data numbers for ArD_0h and ArR_30min were GSE249417 and GSE254935, respectively. Three biological replicates were used for each stage in RNA-seq. The genes with padj ≤ 0.05 and |log2FoldChange| ≥ 1 in the RNA-seq dataset were considered as DEGs. Based on the GO enrichment analysis results of the DEGs from the RNA-seq dataset, genes with transcription factor activity were identified. These TFs were then matched with known TFs identified from the ATAC-seq data to obtain the DETFs.

### 2.4. Co-Expression Network Construction and GO Enrichment Analysis

Based on the FPKM values of the genes from the RNA-seq results, a co-expression analysis was performed between the DETF genes and the DEGs identified in the RNA-seq [27]. The co-expression relationships between the DETF genes and DEGs were determined using the Spearman correlation coefficient (SCC) by employing the cor.test function in the R base packages. Co-expression relationships where |SCC| ≥ 0.9 and *p*-value ≤ 0.01 were used to construct the co-expression network. A positive SCC indicated a positive regulatory relationship between the transcription factor and the gene, while a negative SCC indicated a negative regulatory relationship. Cytoscape [30] was used to create basic co-expression networks. GO enrichment analysis of the DEGs was implemented by GOseq (version 4.10.2, Parkville, Australia) [31].

### 2.5. Artemia Hatching and Culture

*A. parthenogenetica* were cultured in 30‰ artificial seawater under a photoperiod of 16 h of light and 8 h of darkness per day. To induce the post-diapause stage, diapause cysts were treated with dehydration and exposure to −20 °C. For embryo activation and to promote continuous development, the dry cysts were fully rehydrated by hatching in 30‰ artificial seawater (Blue Starfish, Hangzhou, Zhejiang, China) at 28 °C under continuous illumination, marking the beginning of the EDT process in *Artemia*. There must have been some differences between the laboratory conditions and natural conditions to activate the diapause cyst. For example, the temperature changes under natural conditions while the laboratory promoted the development of the diapause embryos through a constant temperature. However, simulating the termination conditions of diapause under natural conditions was tried in the laboratory to minimize the influence of environmental factors on the expression of genes.

For both the diapause stage and 30 min after EDT, three distinct cyst samples from each stage were quickly collected, immediately frozen in liquid nitrogen, and stored in a −80 °C freezer for subsequent RT-qPCR experiments. The diapause stage samples were labeled as ArD_0h (ArD_0h_1, ArD_0h_2, ArD_0h_3), while the samples collected 30 min after EDT were labeled as ArR_30min (ArR_30min_1, ArR_30min_2, ArR_30min_3).

### 2.6. RT-qPCR Verification of DETF

An 80 mg sample with individual cysts grouped together was used for each RT-qPCR experiment. Three biological replicates were used for each gene. RNA was extracted with TRIzol reagent (Invitrogen, San Diego, CA, USA) according to the manufacturer’s instructions; the total RNA (1 μg) was reverse transcribed in a 10 μL reaction containing oligo (dT) primers and M-MLV Reverse Transcriptase (TaKaRa, Shiga, Japan). PCRs were performed using SYBR Green (TOYOBO, Osaka, Japan) on the Bio-Rad MiniOpticon real-time PCR system in triplicate. The housekeeping gene utilized was tubulin. The list of primers used for real-time qPCR analysis of the cysts are shown in Supplementary File S1.

### 2.7. Expression of the Target Genes from 0 h to 5 h After EDT

This was performed in order to observe the progression of the genes regulated by the DETFs associated with EDT. The data for the expression of the genes at 5 h after EDT in *A. parthenogenetica* cysts were further obtained from the GEO database, with the data number listed as GSE249417. The expression of the target genes for the DETFs were extracted from the 5 h dataset and compared with that from 0 h and 30 min to examine the changes in gene expression from 0–5 h after EDT.

## 3. Results

### 3.1. Enriched Motif Analysis and Functional Analysis of ATAC Peaks in Diapause and Activated Cysts

Motif identification revealed motifs of lengths 8, 10, 12, and 14 in both diapause and EDT samples. After eliminating duplicates from the significantly enriched motifs of various lengths in each sample, the final set of enriched motifs was obtained. These enriched motifs were then subjected to enrichment analysis by comparing them with the motifs of known TFs, leading to the identification of corresponding transcription factors for each sample. Since a single motif can be associated with multiple known TFs, the number of matched known TFs far exceeded the number of enriched motifs. Table 1 shows the number of enriched motifs and identified known TFs for each sample. The detailed information of the enriched motifs can be found in Supplementary File S2 and the results of the enrichment analysis to known TFs can be found in Supplementary File S3.

### 3.2. Identification of EDT Related TFs

Although hundreds of TFs were identified in both the diapause and EDT samples, further analysis of the differential gene expression was needed to determine whether these TFs were related to the EDT process of Artemia. Transcriptome sequencing revealed 650 DEGs in the comparison between ArR_30min and ArD_0h (Supplementary File S4). To further identify potential differentially expressed TFs, DEGs with transcription factor activity were screened based on the GO annotations. These DEGs were found to be enriched in four GO items related to transcription factor activity: GO:0000988 (transcription factor activity, protein binding), GO:0000989 (transcription factor activity, transcription factor binding), GO:0001071 (nucleic acid binding transcription factor activity), and GO:0003700 (transcription factor activity, sequence-specific DNA binding). Finally, a total of eleven genes with transcription factor activity were enriched within these GO items, of which ten were upregulated and one was downregulated. The number of enriched DEGs and matched TFs for each GO item are shown in Table 2. When these genes were matched with the TFs identified through ATAC-seq analysis across the samples, four differentially expressed transcription factors (DETFs) were identified: Nuclear Receptor Seven Up (SVP), Proto-oncogene c-Myc (MYC), Retinoid X receptor (RXR), and Mothers against decapentaplegic homolog 6 (SMAD6). These genes not only showed differential expression but also had differentially enriched peaks in their upstream regulatory regions. Their encoding proteins were considered as the TFs functioning in the EDT process.

### 3.3. Transcriptional Regulatory Network of the EDT Process in Artemia

**Figure 2:**
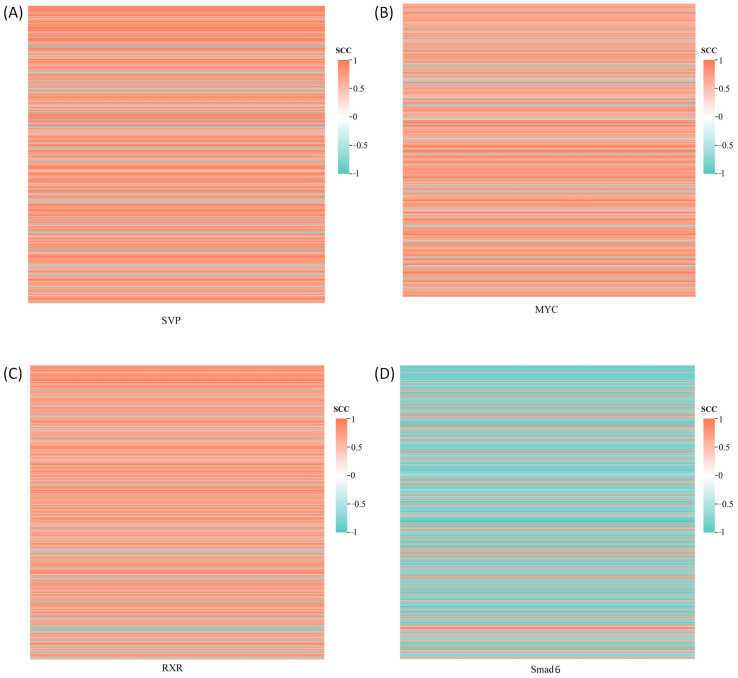
Co-expression analysis of DETF and DEGs. SCC: Spearman correlation coefficient. Each row represents a DEG. (**A**) Co-expression of DEGs with SVP; (**B**) Co-expression of DEGs with MYC; (**C**) Co-expression of DEGs with RXR; (**D**) Co-expression of DEGs with Smad6.

**Figure 3:**
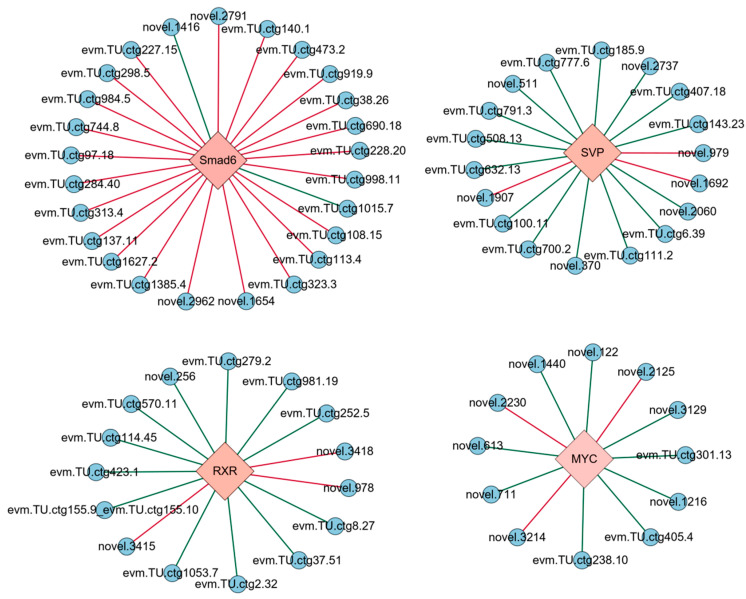
Transcriptional regulatory network of the EDT process in *Artemia*. TF was represented by pink diamonds and their target genes were colored with blue circles. The green line stands for positive regulation; the red line stands for negative regulation.

The co-expression analysis was conducted between four DETFs and 650 DEGs identified from the transcriptome, with the results displayed in Figure 2. Co-expression relationships meeting the thresholds of |SCC| ≥ 0.9 and *p*-value ≤ 0.01 were selected to construct a transcriptional regulatory network linking the TFs with their target genes (Figure 3). The genes regulated by the four DETFs performed distinct biological functions. The target genes of SVP were predominantly involved in cell adhesion and signal transduction, with evm.TU.ctg777.6 (*WNT7B*) participating in the Wnt signaling pathway. Additionally, the target gene evm.TU.ctg6.39 (*AGPAT5*) exhibited transferase activity to transfer phosphorus-containing groups. MYC regulated the target gene evm.TU.ctg155.9_evm.TU.ctg155.10 (*DMD*), which plays a role in protein binding. The target gene evm.TU.ctg155.9_evm.TU.ctg155.10 (how) of RXR was involved in RNA binding, while evm.TU.ctg1053.7 (*UCK2*) showed transferase activity to transfer phosphorus-containing groups. Smad6 regulated genes involved in signal transduction, cell adhesion, and oxidation–reduction processes. The DETF-regulated genes along with their GO annotations are provided in Supplementary File S5.

### 3.4. RT-qPCR Verification of DETFs

**Figure 4:**
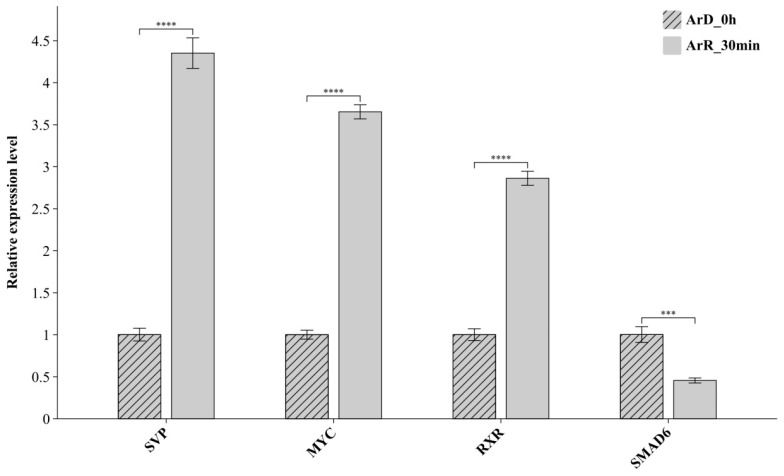
The results of RT-qPCR for the DETFs. ‘***’ represents the *p* < 0.001 significance level; ‘****’ represents the *p* < 0.0001 significance level.

The expression of the DETFs between the ArR_30min and ArD_0h datasets were further verified with RT-qPCR. The results are shown in Figure 4. Based on the RT-qPCR results, SVP, MYC and RXR were upregulated, and SMAD6 was downregulated, which was completely consistent with the integration analysis of the ATAC-seq and RNA-seq datasets. The results of the RT-qPCR further verified the significant differential expression of the DETFs and also confirmed the reliability of the integrated analysis based on the two high-throughput sequencing techniques.

### 3.5. Expression of the Target Genes from 0 h to 5 h After EDT

**Figure 5:**
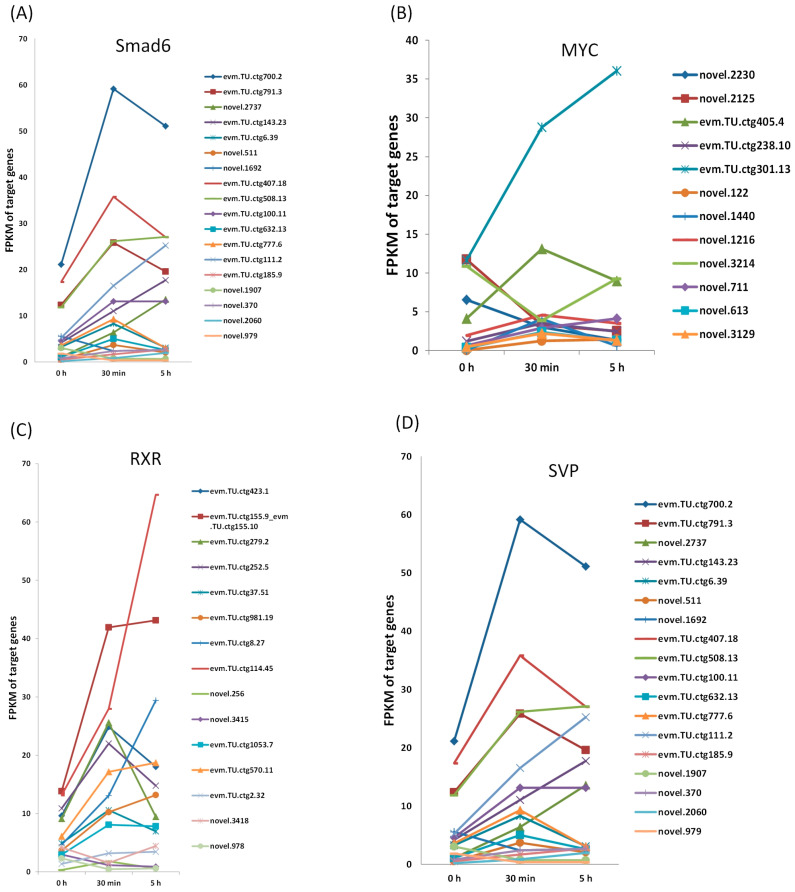
Expression of the target genes for the DETFs at 0 h, 30 min, and 5 h after EDT. (**A**) Expression of the target genes for Smad6; (**B**) Expression of the target genes for MYC; (**C**) Expression of the target genes for RXR; (**D**) Expression of the target genes for SVP.

In order to observe the progression of the target genes for the DETFs during EDT, their expression at 5 h after EDT was obtained from the GEO database. The alteration of gene expression was observed to reach the peak at 5 h after EDT at the transcription level [32]. The expression of the target genes for the four DETFs at 0h, 30 min, and 5 h after EDT are shown in Figure 5. For most target genes, their expression at 30 min and 5 h varied wildly, which was consistent with our previous study [27]. This further proved that the regulation mechanism before and after the first 30 min of EDT might be quite different. This may be because EDT is a continuous process. At different stages, the progress of embryonic development is variable. Therefore, the gene expression at different time points vary considerably. This also indicated that the first 30 min is an important time point for studying the activation of EDT.

In this work, a transcriptionally regulated network association with EDT was constructed based on the ATAC-seq and RNA-seq datasets, which included four TFs and 70 target genes. The TFs functioned in different biological processes. The expression of these TFs was validated with the RT-qPCR experiment and the further exploration on the progression of their target genes from 0–5 h after EDT demonstrated that the first 30 min is an important period for the activation of the EDT process.

## 4. Discussion

### 4.1. TFs in the EDT Process of Artemia

ATAC-seq is used to assess the chromatin accessibility in the upstream regulatory regions of genes. Since these regions contain TF binding sites, this method offers valuable insights into the identification of TFs. By integrating TFs enriched through ATAC-seq with gene expression profiles from RNA-seq, co-expression analysis can reveal the TFs involved in the EDT process and their target genes. In this study, the combined analysis of ATAC-seq and RNA-seq identified four TFs associated with the EDT process. Among these, SVP, MYC, and RXR were upregulated, while SMAD6 was downregulated. Despite the downregulation of SMAD6 at 30 min post-EDT, this did not negate its role in the process. On the contrary, co-expression analysis indicated that SMAD6 negatively regulated most of its target genes. The reduced expression of SMAD6 during EDT led to the upregulation of these genes, enabling them to perform their respective functions. Therefore, the regulatory roles of TFs should be assessed based on both their expression levels and their interactions with target genes to fully understand their role in EDT. Furthermore, all four of the TFs exhibited both positively and negatively correlated target genes, suggesting that these transcription factors are involved in both the diapause and EDT processes, with different target genes expressed at different stages.

SVP has been reported to play a role in regulating insect development and metamorphosis. It plays crucial roles in both the larval–pupal and pupal–adult metamorphoses of *Tribolium castaneum* [33]. SVP influences the synthesis of juvenile hormone in both *Aedes aegypti* and *Blattella germanica* by inhibiting the action of 20-hydroxyecdysone [34,35,36]. It has not been found to function in the diapause or EDT processes before. The result of this work may indicate a new diapause-related function of SVP.

MYC has been found to be critical in the diapause-like state of embryonic stem cells. The depletion of MYC causes the embryo to enter diapause and is associated with the downregulation of biosynthetic pathways in diapause, although its mechanism is still poorly understood [18]. In this study, among the twelve target genes regulated by MYC, eight showed high expression during the EDT stage, while three exhibited elevated expression during the diapause stage.

RXR and EcR are known to form dimer complexes in crustaceans with methyl farnesoate, a sesquiterpenoid hormone analogous to the insect juvenile hormone III that plays a key role in regulating the activation and binding affinity of the RXR/EcR dimer [37]. The expression of RXR was upregulated during the EDT process of *C. finmarchicus* [38], which is consistent with the upregulation of RXR at 30 min after EDT shown in this study.

SMADs were shown to play a role in the hypometabolic response of anoxia and dehydration through the regulation of cell cycle arrest, angiogenic processes, and oxidative injury management strategies [39]. In addition, SMADs were found to play important roles in the control of the dauer/non-dauer switch in *C. elegans* [40]. In this study, SMAD6 was found to function in both the diapause and EDT processes of *Artemia* embryos, with more target genes upregulated in EDT process.

### 4.2. Function of the TFs in the EDT Process of Artemia

The functional analysis of genes regulated by DETFs allows for the identification of the roles these TFs play. The target genes of these TFs are different, indicating that they may exert unique functions in the EDT process by regulating different genes. Based on the functions of the target genes, SVP may regulate cell adhesion, signal transduction, and the transfer of phosphorus-containing groups. MYC may play a role in the regulation of protein binding. RXR might be involved in the regulation of RNA binding and transfer of phosphorus-containing groups. Smad6 possibly regulates the signal transduction, cell adhesion, and oxidation–reduction processes. These results indicate that the cell adhesion, signal transduction, the transfer of phosphorus-containing groups, protein binding, RNA binding, and oxidation–reduction processes might be associated with diapause termination. In addition, although the functions of the DETFs vary differently, some of their target genes share some functional similarities, which may allow indirect associations between different DETFs. For example, four DETF-regulated target genes are involved in signal regulation and transduction, including SVP’s target gene evm.TU.ctg777.6 (*WNT7B*) and three target genes of SMAD6: evm.TU.ctg108.15 (*Tollo*), evm.TU.ctg1627.2 (*INPP5B*), and evm.TU.ctg473.2 (*raskol*). *WNT7B* encodes a ligand for the members of the Frizzled family of seven-transmembrane receptors, playing a key role in the canonical Wnt/β-catenin signaling pathway. It is essential for CNS angiogenesis and blood–brain barrier regulation in vertebrates [41]. *Tollo* encodes the Toll-related receptor TOLL8, which may participate in the Dpp signaling pathway to determine the proximal cell fate in *Drosophila* wings [42]. *INPP5B* encodes a type II inositol 1,4,5-trisphosphate 5-phosphatase (I5P2), responsible for hydrolyzing phosphatidylinositol 4,5-bisphosphate (PtIns(4,5)P2) and the signaling molecule phosphatidylinositol 1,4,5-trisphosphate (PtIns(1,4,5)P3), thus influencing cellular signaling events [43]. *Raskol* encodes a Ras GTPase-activating protein, which acts as a negative regulator for some members of the Ras family [44]. In this study, these signal transduction-related genes were all upregulated during the EDT stage, suggesting that they may be involved in signal regulation during the EDT process of *Artemia*. In addition, not all of the functions of the target genes are annotated. The functions of the target genes with unclear annotations need to be further verified in further research, which may supply more understanding about the TFs related to these genes.

Embryonic diapause is an adaptive survival strategy that allows for a better understanding of how animals respond to environmental changes by studying its mechanisms. This has significant guiding implications for agricultural production and ecological protection, aiding farmers and ecologists in formulating more effective management and conservation measures. Furthermore, by exploring the molecular mechanisms of embryonic diapause, more additional physiological processes might be uncovered, such as the stress response mechanism, thereby advancing biotechnology research in related fields.

The exploration of the TF functions in this work is not exhaustive, primarily due to the lack of clear functional annotations for some target genes. The functions of these genes still need to be validated through further experiments. With comprehensive gene annotation, the roles of the TFs in EDT can be clarified, and a deeper analysis of the different response mechanisms of *Artemia* in adverse or favorable environments can be conducted. This work is of great significance for studying its interactions with the environment and the mechanisms of stress resistance in animals.

## 5. Conclusions

Dormancy is a widely existing environmentally adaptive physiological phenomenon in the biological world. Current research on the transcriptional regulation of *Artemia* diapause has predominantly focused on the initiation and maintenance of diapause, with limited studies on the EDT process. In this study, four key TFs and their target genes in the EDT process of *Artemia* were explored through the integration analysis of ATAC-seq and RNA-seq datasets. The expression of the key TFs was verified by RT-qPCR. These TFs play distinct roles in the EDT process, including cell adhesion, signal transduction, and oxidation–reduction processes. The results of this study provide essential insights for further investigation into the transcriptional regulation mechanisms of EDT in *Artemia*. Given that the diapause mechanism may have a high degree of conservation, the research results of this article also have reference value for the study of dormancy mechanisms in other organisms.

## Figures and Tables

**Table 1:** Number of enriched motifs and identified TFs.

Sample	Number of Enriched Motifs	Number of Known TFs
ArD_0h_1	33	154
ArD_0h_2	39	276
ArD_0h_3	36	248
ArR_30min_1	35	456
ArR_30min_2	42	317
ArR_30min_3	44	404

**Table 2:** Number of enriched DEGs and matched TFs in TF-related GO items.

GO Items	Description	Enriched DEGs	Matched TFs
GO:0000988	transcription factor activity, protein binding	9	1
GO:0000989	transcription factor activity, transcription factor binding	9	1
GO:0001071	nucleic acid binding transcription factor activity	37	10
GO:0003700	transcription factor activity, sequence-specific DNA binding	37	10

## Data Availability

The data are contained within the article and Supplementary Files. The original contributions presented in the study are included in the article and Supplementary Files; further inquiries can be directed to the corresponding author.

## Supplementary Materials

The following supporting information can be downloaded at: https://www.mdpi.com/article/10.3390/genes16020175/s1. Supplementary File S1: Primer sequences used for RT-qPCR. Supplementary File S2: Information for enriched motifs in each sample. Supplementary File S3: Results of enrichment analysis for known TFs in each sample. Supplementary File S4: The DEGs between ArR_30min and ArD_0h. Supplementary File S5: The DETF-regulated genes along with their GO annotations.

## Abbreviations

Differentially expressed genes (DEGs). Differentially expressed transcription factor with significant chromatin binding motifs G-protein-coupled receptor (DETF). Transcription factor (TF). Spearman correlation coefficient (SCC). Embryonic pause termination (EDT).
